# How Long Halt Ye?

**Published:** 1921-01-01

**Authors:** Richard J. Coles

**Affiliations:** Secretary. King's College Hospital, Denmark Hill, S.E. 5


### THE EDITOR'S LETTER-BOX.

**Correspondence n all subjects is invited, but we cannot in any way be responsible for the opinions expressed by our correspondents, who must give their name and address as a guarantee of good faith, but not necessarily for publication. Correspondents are reminded that brevity of style and conciseness of statement greatly facilitate early insertion.**

### "HOW LONG HALT YE?"

*To the Editor of* THE HOSPITAL.

SIR,—With reference to Mr. Glenton-Kerr's letter in last week's issue of THE HOSPITAL, if he will refer to my letter he will find that I did not confine my suggestion as to contributing 2d. per week to the hospitals to either insured persons or to those of the artisan class, but to every salary or wage earner, say over the age of sixteen years. Nor in making my suggestion had I overlooked the existence of Hospital Saturday.

A vast number of those whom Mr. Glenton-Kerr refers to as weekly wage earners do support the hospitals; on the other hand, viewing the matter from a broad point of view, it is inaccurate to say that the vast majority do contribute generously to hospital funds.

The fact that only about 10 per cent. of the population supports the hospitals of London is at variance with Mr. Glenton-Kerr's contention. It is *not to any one class* we must look for support for our hospitals, but to *every single person earning*, irrespective of the nature of employment, professional or otherwise, and both male and female.

When every worker, professionally or otherwise, has decided to support our hospitals in London and has requested his or her employer to retain the amount which he or she has determined to contribute weekly, monthly, or otherwise, and every employer has agreed to do this and to contribute a similar amount in respect of each employee, then, and only then, can it be said that both employers and employed are doing what they can to support the hospitals. And when such a scheme has been achieved more convalescent accommodation can be provided, more bed accommodation will be available in the hospitals by the greatly reduced average stay per patient, the waiting-lists will be reduced to a minimum, if not wiped out, and the hospitals will be financially strong.

I do not in any way belittle the great work which the Hospital Saturday Fund is doing, and I will gladly do anything I can to assist the Fund and all connected therewith to achieve even greater results.

Again, I say, I believe that if we organise and put our backs into it we could raise from £2,000,000 to £4,000,000 a year for the hospitals of London.—Yours faithfully,

RICHARD J. COLES, Secretary.

King's College Hospital, Denmark Hill, S.E. 5.
December 20, 1920.

